# Working Remotely During the COVID-19 Pandemic: Work-Related Psychosocial Factors, Work Satisfaction, and Job Performance Among Russian Employees

**DOI:** 10.11621/pir.2022.0101

**Published:** 2022-03-15

**Authors:** Ferdinando Toscano, Eleonora Bigliardi, Marina V. Polevaya, Elena V. Kamneva, Salvatore Zappalà

**Affiliations:** a Department of Psychology, University of Bologna, Italy; b Department of Psychology and Human Capital Development, Financial University under the Government of the Russian Federation, Moscow, Russia

**Keywords:** Remote work stress, remote work engagement, family–work conflict, opinions about remote work, leader–member exchange

## Abstract

**Background:**

The spread of COVID-19 has forced organizations to quickly offer remote work arrangements to employees.

**Objective:**

The study focuses on remote work during the first wave of the pandemic and describes how Russian employees experienced remote work. The research has three main objectives: (1) to investigate the influence of gender and age on employees’ perceptions of remote work; (2) to investigate the relationship between remote work and psychosocial variables, such as remote work stress, remote work engagement, and family–work conflict; (3) to examine whether and how much such psychosocial factors are related to remote work satisfaction and job performance. These objectives were the basis for developing six hypotheses.

**Design:**

A cross-sectional study involved 313 Russian employees. Data were collected using an online survey distributed in April and May 2020. The hypotheses were tested using ANOVA, correlations, and multiple linear regression analyses.

**Results:**

Women experienced more stress and more engagement when working remotely; older employees perceived remote work as a less positive experience; opinions about remote work and remote work engagement were positively related to remote work satisfaction; leader–member exchange (LMX) was a significant predictor of job performance.

**Conclusion:**

During the lockdown, remote work was perceived as a positive experience. We discuss some practical implications for organizations and managers.

## Introduction

The health emergency caused by the spread of the new coronavirus (COVID-19) forced organizations and employees to rapidly adopt flexible work arrangements with the primary goal of slowing down the diffusion of the infection.

A report published by Ernest & Young (27 March 2020) showed that, during the first wave of the COVID-19 pandemic, all the interviewed Russian employers adopted measures that changed their way of working, and 97% of companies partially or fully implemented remote work programs. The switch to remote work was the primary adjustment that Russian organizations used to respond to the health crisis. The adoption of this new arrangement was a challenge, because it was necessary to introduce some changes to work procedures and to Russian legal regulations, which only partially supported this type of work. In 53% of cases, remote work was implemented through an additional agreement between employers and staff. In 32% of organizations, a new contract was requested, requiring additional effort for the organizations and their representatives. The health emergency raised the need for a step forward in the legislation. On 8 December 2020, Federal Law No 407-FZ amended the Russian Labor Code, introducing remote work and equating it with the pre-existing concept of “distance work”.

The scientific literature describes such work in many ways. One of the most common terms is teleworking, which is defined as “an alternative work arrangement in which employees perform tasks elsewhere that are normally done in a primary workplace, for at least some portion of their work schedule, using electronic media to interact with others inside and outside the organization” (Gajendran & Harrison, 2007, p. 1525). Other terms such as remote work, nomadic working, agile working, and homeworking (Groen, van Triest, Coers, & Wtenweerde, 2018; Karia & Asaari, 2016) are often used interchangeably with it. Despite differences in terminology, these names all emphasize the absence of a stable workplace and the use of information and communication technologies (ICTs). This paper uses the term “remote work” to describe the working from home that many Russian employees experienced during April and May 2020.

During the lockdown and subsequent phases of the health emergency, the pandemic forced employees to transfer to their homes as much as possible of the work regularly done in the office. Such arrangements made working in this period a bit different from the remote work experience of the past, when employees alternated working at the office and in other places. The pervasiveness of the technological component made, instead, this work from home similar to the remote work experienced in the past by employees.

Over the years, many authors have studied the consequences of remote work for employers and employees (Groen et al., 2018). Baruch (2000) suggests that remote work may have consequences for a) identity (self-concept as teleworker and change in the family role); b) skills (the development of time management skills); c) context (control of environmental distractions); d) role demands (demands and constraints related to job activities and social relationships); and e) role outcomes (changes in job satisfaction and job performance).

Mainly focusing on this last type of outcome, this study offers an overview of the experience of a sample of Russian remote workers during the first wave of the pandemic. In particular, this research contributes to: a) assess whether some sociodemographic variables (gender and age) affect Russian employees’ perceptions about the psychosocial experience of remote work (opinions about remote work, remote work engagement, remote work stress, work–family conflict); b) evaluate whether the percentage of office activities accomplished at home and the extent of goal formalization affect these psychosocial factors; c) test whether the aforementioned psychosocial factors and the relationship of employees with their supervisor (leader–member exchange, LMX) are related to employees’ remote work satisfaction and job productivity.

In the next section, we explore the influence of gender and age on employees’ perceptions of remote work. Then, we describe the relationship between remote work and different psychosocial variables, such as opinions about this way of working, remote work stress and engagement, and family–work conflict. Finally, we review the literature on predictors of remote work satisfaction and general job performance in order to clearly express the purposes of our study and then to show and comment on its results.

## Literature Review and Hypotheses

### Gender, Age, and the Experience of Remote Work

Various studies have investigated the association between socio-demographic variables and telework behavior (Drucker & Khattak, 2000; Parasuraman & Simmers, 2001; Sener & Bath, 2011; Zhang, Moeckel, Moreno, Shuai, & Gao, 2020). Scholars found that flexible work was more attractive for females than males because it decreased work–family conflict, that is, the interference of work commitments over family commitments (Parasuraman & Simmers, 2001). Such evidence confirmed remote work as the perfect solution for combining work and family demands (Huws, 1996).

Home-based teleworking, however, should not be considered the remedy for all the difficulties that women experience when trying to arrange family life and work. In fact, remote work may reinforce the gendered division of labor (Sullivan & Lewis, 2001) and increase men’s expectations that women should take care of family work (Hartig, Kylin, & Johansson, 2007; Toscano & Zappalà, 2020a). Accordingly, since women tend to dedicate more time and energy to household chores and childcare responsibilities than their partners, remote work may lead women to an increased perception of stress and isolation (Parasuraman & Simmers, 2001; Weinert, Maier, & Laumer, 2015).

On the other hand, the research also highlights that females are more motivated to work remotely by its flexibility, convenience, and autonomy. Therefore, lowering the conflict and increasing flexibility and autonomy may enhance women’s motivation to engage with remote work (Chapman, Sheehy, Heywood, Dooley, & Collins, 1995). This consideration, combined with the previously stated research findings, leads us to hypothesize that:

*Hypothesis 1*: During the remote work experienced in the lockdown, women were more engaged and had more positive opinions about remote work, but they were also more stressed and perceived greater family–work conflict than men.

The relationship between age and telework has been extensively investigated by scholars. Young and older workers constitute groups that experience both advantages and disadvantages of remote work. For example, older workers are considered more frequently trustworthy, reliable, independent, and able to manage time, and therefore more suitable for remote work. On the other side, they may be less capable of adjusting to changes, especially to new technology, and therefore may be less suitable for remote work (Sharit, Czaja, & Hernandez, 2009). For instance, Sener and Bath (2011) found that employees over 30 years old can exercise personal choices regarding work arrangements and goal setting, while younger employees are more accustomed to working on-site in order to create relationships with colleagues and supervisors. Therefore, according to this study, young people would be less interested in remote work than older ones.

In contrast, according to Drucker and Khattak (2000), younger workers generally prefer to work from home because they are more comfortable using ICTs than are older workers. On the other hand, a study conducted in the academic context of an advanced post-Soviet country found no significant age differences in telework use (Arvola & Kristjuhan, 2015).

Despite the conflicting results, younger generations place greater importance on work–family balance than their older colleagues. They use telework to improve their quality of life and reduce conflict with their partner (Kwon & Jeon, 2017). At the same time, a recent study by Raišienė, Rapuano, Varkulevičiūtė, and Stachová (2020) found that older workers emphasized the disadvantages of telework. They preferred more contact with managers and colleagues, faced several difficulties in self-organization, and perceived higher work–family conflict. In contrast, their younger colleagues mostly recognized that work flexibility required specific skills and competencies. Therefore, based on previous literature, we hypothesize the following:

*Hypothesis 2*: During remote work experienced in the lockdown, age was positively related to remote work stress and negatively related to opinions about remote work, leader–member exchange, remote work engagement, and family–work conflict.

### Percentage of Office Activities Done Remotely and Remote Work Goals

An important area in the study of remote work pertains to the practical aspects of teleworking, such as the number of office activities carried out at home or the modalities used to define work goals. However, despite the importance of these remote work characteristics, research has not yet focused much attention on these aspects.

In this study, the amount of activities performed remotely indicates the percentage of tasks usually performed at the office that employees were able to perform from home (which we defined as “percentage of office activities at home”). Our conceptualization considers how much the office work was reorganized to be performed at home during the health emergency.

Two studies treated the number of remote activities in terms of hours spent working remotely, finding that too much teleworking may have harmful effects (Golden & Veiga, 2005; Virick, DaSilva, & Arrington, 2010). However, although a later study partially disproved their thesis (Vander Elst et al., 2017), these authors claimed that there is a curvilinear relation between the extent of remote work and job satisfaction: at low levels of remote work, individuals are still capable of taking advantage of social interactions with colleagues, which enhances their job satisfaction; conversely, large amount of remote work increases social isolation, which decreases job satisfaction (Toscano & Zappalà, 2020b).

In the case of the COVID-19 pandemic, many employees were forced to perform their work activities only from home. There is little evidence of the effects of substituting direct contact among colleagues with communication via the Internet (Fonner & Roloff, 2012). On the other hand, the introduction of communication systems increasingly similar to face-to-face interaction, made possible by advances in technology (e.g., video call programs), may reduce social isolation by increasing social participation (Baker et al., 2018; Kato, Shinfuku, & Tateno, 2020).

Due to the absence of specific studies on the subject, there is limited evidence on the amount of “usual office activities” carried out in remote work. There is only some evidence on the amount of weekly time spent working remotely. We hypothesize that employees having an adequate amount of activities to carry out remotely experience the same psychological effects when they work the same amount of time (in terms of hours per week) in the office. In other words, we expect that the possibility to carry out remotely the same amount of work done in the office may have positive consequences for homeworkers, such as more engagement and less stress. Therefore, we hypothesize that:

*Hypothesis 3:* During remote work experienced in the lockdown, the percentage of office activities was positively related to opinions about remote work, remote work engagement, and leader–member exchange, and negatively related to remote work stress and family–work conflict.

Goal formalization in remote work during the pandemic was also a focus of our attention. This study considers how supervisors and managers established goals that employees had to pursue when working at home. Setting goals is critical for increasing job performance because it leads employees to act according to the goal’s requirements (Locke, Frederick, Lee, & Bobko, 1984).

We distinguished three degrees of goal formalization: a) undefined goals, b) orally defined goals, and c) written goals. Although the previous literature did not study the differences among these modalities, we conceive written goals as the most advanced way, since they give employees a clear guideline, clarify when they reach the goal and, in the end, improve their engagement toward work goals.

Research highlights the importance of setting goals as a functional and motivational practice (Locke & Latham, 2006), but less attention has been given to its implementation modalities. To solve this theoretical gap, we hypothesize that setting written goals has positive outcomes for homeworkers, since they can check objectives at any time and set sub-goals to organize their work. Thus, we hypothesize the following:

*Hypothesis 4:* During remote work experienced in the lockdown, the formalization of work goals was positively associated with opinions about remote work, remote work engagement, and leader–member exchange, but negatively associated with remote work stress.

### Predictors of Remote Job Satisfaction and Job Performance

The scientific literature suggests that remote work is generally related to higher job satisfaction and job performance (Allen, Golden & Shockley, 2015; Toscano & Zappalà, 2020a).

Gajendran and Harrison (2007) observed that telework was positively related to employees’ job satisfaction. As mentioned above, previous studies (Golden & Veiga, 2005; Virick et al., 2010) found a curvilinear relation between remote work and job satisfaction, and that remote work is positively related to job satisfaction when there are lower levels of teleworking. Remote work satisfaction—that is, the satisfaction experienced for work done remotely—is also positively related to good relationships in the workplace (e.g., with colleagues; Allen, et al., 2015), organizational support, and leader–member exchange (Baker, Avery, & Crawford, 2007; Golden, 2006). Furthermore, remote work is perceived as a positive experience, and when employees are highly engaged in remote work, they also have a reasonable opinion of it (Derks, van Duin, Tims, & Bakker, 2015). Therefore, in line with previous literature (Nakrošiene, Buciuniene, & Goštautaite, 2019), we argue that engagement and positive opinions about remote work are positively related to satisfaction with remote work and, according to the previous results, we hypothesize that:

*Hypothesis 5:* During remote work experienced in the lockdown, remote work satisfaction was positively related to remote work engagement, opinions about remote work, and LMX, and negatively related to remote work stress and family–work conflict.

Previous studies examined several variables affecting the relationship between remote work and job performance. For example, Gajendran and Harrison (2007) argued that relationships with colleagues and supervisors influence job performance. In particular, LMX enhances productivity and supervisors’ evaluation. Breevaart, Bakker, Demerouti, and van den Heuvel (2015) demonstrated that work engagement is also positively associated with job performance, because vigor, dedication, and absorption (the components of work engagement) allow employees to work with energy and enthusiasm, and to be absorbed in the work tasks. Therefore, even during a lockdown, high levels of remote work engagement may ensure good job performance.

On the contrary, family–work conflict and job stress should be associated with decreased job performance. Among the numerous studies on this aspect, Netemeyer, Maxham, and Pullig (2005) found a negative relationship of family–work conflict with both in-role performance and extra-role performance as evaluated by supervisors. Analogous results have been confirmed also by recent studies (e.g., Nohe, Michel, & Sonntag, 2014). Although these relationships were less investigated in a remote work context, we argue that comparable results should be observed in remote work during the lockdown, especially considering that governments mandated it and that there was limited opportunity to prepare employees to work remotely. In other words, we argue that the family–work conflict experienced during the lockdown may decrease job performance. Therefore, based on previous literature, we hypothesize that:

*Hypothesis 6:* During remote work experienced in the lockdown, job performance was positively related to remote work engagement, opinions about remote work, and LMX, and negatively related to remote work stress and family–work conflict.

## Methods

### Procedures

The current study has a cross-sectional design. Data were gathered through an online survey administered through the platform Qualtrics, distributed in April and May 2020 and aimed to investigate the experience of homeworking during the first wave of the pandemic in Russia.

The study used a convenience sampling technique based on voluntary participation. To follow the Declaration of Helsinki concerning ethics in research, complete confidentiality of individual responses was guaranteed, and the questionnaires were collected anonymously.

### Participants

The sample consisted of 313 participants, 219 (70%) of whom were females. Fifty percent of respondents were between 36 and 55 years old. Length of service ranges between 0 and 34 years old, with a mean value of 7.6 years. Half of the participants (50%) had a Master’s Degree or a Ph.D. Most respondents work in the tertiary sectors: 143 (45.7%) in the private sector, 134 (42.8%) in the public sector, and the remaining 10% in the primary or secondary sector. The majority of the sample (64%) was composed of employees, while 34% were supervisors, managers or middle managers.

### Measures

The survey was composed of different sections: 1) the first investigated opinions and experiences about remote work during COVID-19; 2) the second investigated opinions and experiences about one’s work in general and, 3) the third included socio-demographic information. Except where otherwise specified, the study used a 5-point Likert-type response scale ranging from 1 (completely disagree) to 5 (completely agree). All items were administered in the Russian language.

#### 1) Opinions and Experiences About Remote Work

The percentage of office activities (tasks usually performed at the office) that employees performed at home was measured with a single item. The possible answers were: less than 25%, between 25% and 50%, between 50% and 75%, and more than 75%.

Goal formalization: a question examined how supervisors defined task goals during the lockdown. The answer options were “not defined at all”, “defined orally” or “defined in writing”.

Remote work stress was measured using the 4-item scale developed by Weinert et al. (2015). An example of an item is “I feel exhausted working from home”.

Remote work engagement was measured using the ultra-short 3-item scale validated by Schaufeli, Shimazu, Hakanen, Salanova, and De Witte (2019). Examples of items are “When working remotely: 1) I feel full of energy; 2) I am excited about my work”.

Family–work conflict was measured using three items of the scale developed by Netemeyer, Boles, and McMurrian (1996). An example of an item is “The demands of my family or partner interfere with my work activities”.

Opinions about remote work were investigated using the 4-item scale developed by Staples, Hulland, and Higgins (1999). Examples of items are “Remote work is not a productive way of working” and “Remote work is difficult”.

Remote work satisfaction was measured using three items derived by Lee and Brand (2005) and previously used by Toscano and Zappalà (2020b). Two examples of an item are “Once the emergency is over, if I had to decide to work remotely again, I would choose it” and “If a friend asked me if it is appropriate to work remotely, I would recommend it”.

#### 2) Opinions and Experiences About One’s Work

Leader–member exchange (LMX) was measured using the 7-item scale developed by Graen and Uhl-Bien (1995). An example of an item is “My leader recognizes my potential”.

Job performance was measured using a 6-item scale developed by Staples et al. (1999). Examples of items are “I believe I am an effective employee” and “I am happy with the quality of my work output”.

#### 3) Socio-Demographic Measures

Finally, we recorded gender (1 = male, 2 = female), and age (six age ranges were provided; 1: less than 25 years old, 2: 26–35; 3: 36–45; 4: 46–55; 5: 56–65; 6: over 65).

### Data Analysis

Before testing the hypotheses, Cronbach alphas, means, and standards deviations were computed. ANOVA was used to test hypothesis 1, and correlations and linear regressions were used to test, respectively, hypotheses 2–4 and hypotheses 5 and 6. All the analyses were carried out using SPSS 26.

## Results

Mean values, standard deviations, bivariate correlations, and Cronbach alphas are presented in *Table 1*. Scores higher than the mean value were observed for several variables and suggest, for instance, that employees were generally engaged and satisfied with the experience of remote work and had a reasonable opinion of their remote work experience during the lockdown.

**Table 1:** Means, standard deviations, alphas, and bivariate correlations among the study variables

	*M*	*SD*	1.	2.	3.	4.	5.	6.	7.	8.	9.	10.	11.
1. Gender	1.70	0.46	–										
2. Age	2.97	1.24	.05	–									
3. Percentage of office activities	3.46	0.86	–.01	–.12	–								
4. Goal formalization	2.86	0.60	.03	–.05	.10	–							
5. Remote work stress	2.87	1.39	.22**	.03	–.10	.09	*(.92)*						
6. Remote work engagement	3.08	0.95	.11	–.15*	.27**	.15*	–.39**	*(.69)*					
7. Family–work conflict	2.29	1.25	.05	.02	–.07	.01	.42**	–.28**	*(.81)*				
8. Opinions about remote work	2.97	1.07	–.04	–.15*	.29**	.06	–.61**	.57**	–.39**	*(.79)*			
9. LMX	3.85	0.90	.11	–.15*	.11	.04	–.13*	.27**	–.10	.13*	*(.89)*		
10. Remote work satisfaction	3.19	1.32	–.03	–.09	.17**	.07	–.59**	.57**	–.29**	.73**	.15*	*(.90)*	
11. Job performance	4.18	0.69	.12	–.05	.00	.04	–.04	.20**	–.04	.09	.00	.47**	*(.89)*

Note. N = 313. *p < .05. **p < .01, two-tailed tests. In the diagonal, within parentheses, Cronbach’s alphas.

An ANOVA performed to test hypothesis 1 showed that women reported greater perceived stress than men (*M*_w_ = 3.06; *M*_m_ = 2.39; *F* = 13.03, *p <* .01). No gender differences were observed in family–work conflict and opinions about remote work, whereas women showed greater remote work engagement than men, although the difference was only marginally significant (*M*_w_ = 3.15; *M*_m_ = 2.92; *F* = 3.42, *p* = .06). Hypothesis 1 is thus only partially confirmed.

Age was negatively correlated with remote work engagement (*r* = -.15; p < .05), opinions about remote work (*r* = –.15; p < .05), and LMX (*r =* -.15; *p <* .05), thus indicating that older employees experienced remote work less positively. Age was not related to family–work conflict and remote work stress. Thus, hypothesis 2 was partially confirmed.

The percentage of office activities performed remotely was positively related to opinions about remote work (*r =* .29; *p <* .01) and remote work engagement (*r =* .27; *p <* .01). No significant relationships were found with LMX, remote work stress and family–work conflict.

The formalization of the work goals was associated with remote work engagement (*r =* .15; *p <* .05), while it was not related to opinions about remote work, remote work stress, and LMX. Therefore, the observed results confirm only partially hypothesis 4.

Finally, to test the remaining two hypotheses, our independent variables were regressed on remote work satisfaction and remote work job performance. *Table 2* shows that remote work satisfaction was significantly and positively related to remote work engagement (β = .20; *p <* .01) and opinions about remote work (β = .47; *p <* .01), and negatively related to remote work stress (β = –.25; *p <* .01). Since LMX and family–work conflict were unrelated to remote work satisfaction, hypothesis 5 was only partially confirmed. The second regression showed that job performance was significantly and positively related only to LMX (β = .43; *p <* .01), making hypothesis 6 only minimally confirmed.

**Table 2:** Linear regression with remote work satisfaction and job performance as dependent variables

	Remote work satisfaction			Job performance		
	B	SE	β	B	SE	β
1. Remote work stress	–.23**	.05	–.25**	.02	.04	.04
2. Remote work engagement	.28**	.08	.20**	.08	.05	.11
3. Family–work conflict	.02	.05	–.01	.00	.04	–.01
4. Opinions about remote work	.60**	.08	.47**	.00	.05	.00
5. Leader–Member Exchange	–.02	.07	–.02	.34**	.05	.43**
R^2^		.60			.21	
F		64.99**			11.78**	

Note. *p < .05 **p < .01

## Discussion

The current study had the main objective of investigating how Russian employees perceived homeworking during the lockdown. To slow down the spread of the coronavirus, Russian companies rapidly reorganized their way of working, allowing employees to work from home. The results only partially confirmed our six research hypotheses, and five main results could be highlighted in this section.

First, women experienced greater remote work stress and remote work engagement than men. Being more stressed and, at the same time, more engaged with remote work during the lockdown is coherent with previous literature: remote work offered more flexibility and more autonomy to women that increased their remote work engagement (Chapman et al., 1995). On the other hand, the long period of social isolation from the workplace and the increased time dedicated to family needs may have also exacerbated feelings of stress (Wang et al., 2020).

Second, the results highlighted that homeworking was, in general, a worse experience for older employees than for their younger counterparts. As age increased, participants reported lower engagement, less positive opinions about remote work, and lower LMX. This result is coherent with that observed by Drucker and Khattak (2000), who maintain that remote work engagement and opinions may depend on the extent of confidence in the use of ICTs. Older employees are less engaged with remote work probably because they are less accustomed to such technology. Thus, our results confirm the findings by Raišienė and colleagues (2020) that older employees tend to emphasize the disadvantages of telework. Finally, the decrease in social interactions with managers or supervisors might cause a lower LMX.

Even hypothesis 3 was only partially confirmed. The results showed that when employees performed a higher percentage of office activities at home, they also had more positive opinions about remote work and greater remote work engagement. Our results are not in line with those of Golden and Veiga (2005) and Virick et al. (2010), who found that more hours spent teleworking are associated with negative perceptions about it. However, homeworking during the lockdown can be considered a very different situation from teleworking before the pandemic. In a few words, our respondents probably emphasized the importance of carrying out as many office activities as possible at home, as a sort of reassuring substitute for normality that reduced social isolation and kept them busy with daily work tasks.

Fourth, hypothesis 4 was only minimally confirmed, suggesting that having clear and explicit work goals has a limited correlation with individual and work aspects. The extent of goal formalization was associated only with work engagement, which suggests that having clear and written goals (rather than oral or no formal goal at all) can be a significant motivational factor to pursue work goals with time, energy, and effort. Having clear goals and guidelines usually increases the opinion that remote work can be a suitable and valid alternative to office work and improves the relationship with the leader that assigns clear goals and tasks, but this was not the case. According to the goal-setting theory (Locke & Latham, 2006), a possible explanation is that, regardless of whether employees received goals from their supervisor, respondents had their own work goals and used them to drive and monitor their job performance. Furthermore, we cannot exclude that our inconsistent results might be related to the operationalization of the goal formalization variable that we used. Future studies should use a different measure of goal formalization, which might also include self-assigned goals.

Finally, hypotheses 5 and 6 were also only partially confirmed by the multiple regression analyses. In particular, we notice that remote work satisfaction and job performance, as two different outcomes (the first related to remote work and the second related to general work), also have different predictors. Remote work satisfaction was mainly related to individual factors (positive opinions about remote work, remote work engagement, and reduced remote work stress). In contrast, job performance was related to the social-organizational aspect of leader–member exchange. Contrary to our expectations, family–work conflict was not related to remote work satisfaction. One possible explanation is that, when present, family–work conflict might delay or undermine work tasks without affecting satisfaction with remote work as a form of flexible work arrangement. Thus, future studies should better examine whether employees would recommend remote work to others or work remotely themselves regardless of potential interference of family duties with their work. In addition, the patterns we found can be explained by the fact that remote work satisfaction is a subjective experience and thus more related to individual variables connected with remote work (such as remote work engagement or stress). In contrast, job performance is a more general concept that includes both home and office tasks and thus is more related to general work processes, such as LMX. This reasoning might explain why none of the remote work variables was related to job performance.

## Practical Implications

The spread of the coronavirus and the diffusion of remote work pose major challenges to the world of work, in Russia and elsewhere. The findings of this research suggest some practical implications for companies, managers, and human resources experts.

First, the study suggests that organizations and HR officers should consider sociodemographic characteristics when implementing remote work programs. Women and older employees may find impediments in adopting flexible work arrangements, especially at home. The whole society should consider that the workload experienced by women, who have to attend to both remote work and family duties during the pandemic, might cause stress with its adverse effects. Although gender was not related to family–work conflict, the issue should be further investigated. The needs of older employees should also be more thoroughly explored and addressed: older workers showed more reluctance to work remotely than their younger colleagues. Therefore, managers and HR officers should develop practices that support them in switching from office to remote work. Their reluctance might also be related to difficulties using technological tools, so organizations should provide older workers with the necessary skills to manage the devices and software that enable remote work.

An interesting finding of this study is the positive relationship between the percentage of office work performed at home and more positive opinions about homeworking and greater remote work engagement. These results suggest that organizations should establish activities to be implemented outside the office to involve more employees in remote work programs, increase the variety of home tasks, improve employees’ remote work engagement, and set work goals to pursue at home, thus increasing engagement. Organizational performance might eventually be improved by this activity.

Organizations may improve their remote work programs also by empowering the relationships between employees and managers. Trust and the quality of the relationship between managers and workers acquire even more importance and seem to predict job performance. Structuring meetings, as well as virtual events, become very important to maintain a positive relationship. Finally, the literature suggests that, in preparing plans to implement long-term remote work, alternating employees’ presence in the office and at home should be preferred (Golden & Veiga, 2005; Zappalà, Toscano, & Topa, 2021).

## Limitations and Future Research

The contributions of this study should be considered in light of its limitations. First, the research used a cross-sectional design, which limits the possibility of drawing causal inferences. Since the findings of this study may be essential to understand the difficulties of the workforce due to the health emergency, the results should be confirmed using a longitudinal study design. Future research regarding remote work during the COVID-19 pandemic should use this type of research design.

A second concern relates to the sampling technique used for the participants’ selection. The convenience sampling technique based on voluntary participation decreases the generalization of the findings, including because the survey was distributed online and respondents belong to different industries, organizations, and job positions. Future research should involve a sampling technique in which specific contexts (sector or organization) should be addressed. The study also suggests additional research directions. In this research, we introduced two new variables related to the percentage of office activity and how goals are formalized. We emphasize the need for further analyzing these variables, since we consider them very promising. Finally, although we have highlighted the importance of performance and satisfaction, it is important first to assess the performance of remote work and, second, consider remote work performance measured at the group level or using objective criteria.

## Conclusion

The health emergency has extensively changed our way of performing work and considering flexible work arrangements. This study underlines how some aspects can be important for employees who work remotely. On the one hand, psychosocial variables such as remote work engagement and leader–member exchange positively influence, respectively, remote work satisfaction and job performance. On the other hand, employees’ opinions about remote work were more positive when performing a high percentage of office activities at home. This highlights the importance of remote work not only as a parachute for the COVID-19 pandemic, but as a more complex transformation of organizations and their way of working. For this reason, organizations should plan the implementation of remote work with a long-term perspective if they are to observe positive consequences for both organizations and employees.

## References

[ref1] Allen, T.D., Golden, T.D., & Shockley, K.M. (2015). How effective is telecommuting? Assessing the status of our scientific findings. Psychological Science in the Public Interest, 16(2), 40–68. 10.1177/1529100615593273 26403188

[ref2] Arvola, R., & Kristjuhan, Ü. (2015). Workload and health of older academic personnel using telework. Agronomy Research, 13(3), 741–749. 10.1515/saeb-2017-0013

[ref3] Baker, E., Avery, G.C., & Crawford, J. (2007). Satisfaction and perceived productivity when professionals work from home. Research and Practice in Human Resource Management, 15(1), 37–62.

[ref4] Baker, S., Warburton, J., Waycott, J., Batchelor, F., Hoang, T., Dow, B., ... & Vetere, F. (2018). Combatting social isolation and increasing social participation of older adults through the use of technology: A systematic review of existing evidence. Australasian Journal on Ageing, 37(3), 184–193. 10.1111/ajag.12572 30022583

[ref5] Baruch, Y. (2000). Teleworking: benefits and pitfalls as perceived by professionals and managers. New Technology, Work and Employment, 15(1), 34–49. 10.1111/1468-005X.00063

[ref6] Boorsma, B., & Mitchell, S. (2011). Work-life innovation: Smart work – A paradigm shift transforming how, where, and when work gets done. Cisco Internet Business, 1–7.

[ref7] Breevaart, K., Bakker, A. B., Demerouti, E., & van den Heuvel, M. (2015). Leader–member exchange, work engagement, and job performance. Journal of Managerial Psychology, 30(7), 754–770*.* 10.1108/JMP-03-2013-0088

[ref8] Chapman, A. J., Sheehy, N. P., Heywood, S., Dooley, B., & Collins, S. C. (1995). The organizational implications of teleworking. International Review of Industrial and Organizational Psychology, 10, 229–248.

[ref9] Derks, D., van Duin, D., Tims, M., & Bakker, A. B. (2015). Smartphone use and work–home interference: The moderating role of social norms and employee work engagement. Journal of Occupational and Organizational Psychology, 88, 155–177. 10.1111/joop.12083

[ref10] Drucker, J., & Khattak, A. J. (2000). Propensity to work from home: Modeling results from the 1995 Nationwide Personal Transportation Survey. Transportation Research Record, 1706(1), 108–117. 10.3141/1706-13

[ref11] Ernst & Young (2020, March 27). Obzor mer, predprinimaemykh rabotodateliami v sviazi s COVID-19. Retrieved from https://assets.ey.com/content/dam/ey-sites/ey-com/ru_ru/news/2020/03/ey_covid_19_countermeasures_survey.pdf (Accessed on 19^th^ November, 2020).

[ref12] Fonner, K.L., & Roloff, M.E. (2012). Testing the connectivity paradox: Linking teleworkers’ communication media use to social presence, stress from interruptions, and organizational identification. Communication Monographs, 79(2), 205–231. 10.1080/03637751.2012.673000

[ref13] Gajendran, R.S., & Harrison, D.A. (2007). The good, the bad, and the unknown about telecommuting: Meta-analysis of psychological mediators and individual consequences. Journal of Applied Psychology, 92, 1524–1541. 10.1037/0021-9010.92.6.1524 18020794

[ref14] Golden, T.D., & Veiga, J.F. (2005). The impact of extent of telecommuting on job satisfaction: Resolving inconsistent findings. Journal of Management, 31(2), 301–318. 10.1177/0149206304271768

[ref15] Golden, T.D., Veiga, J.F., & Dino, R.N. (2008). The impact of professional isolation on teleworker job performance and turnover intentions: Does time spent teleworking, interacting face-to-face, or having access to communication-enhancing technology matter? Journal of Applied Psychology, 93(6), 1412–1412–1421. 10.1037/a0012722 19025257

[ref16] Golden, T.D. (2006). Avoiding depletion in virtual work: Telework and the intervening impact of work exhaustion on commitment and turnover intentions. Journal of Vocational Behavior, 69, 176–187. 10.1016/j.jvb.2006.02.003

[ref17] Groen, B.A., van Triest, S.P., Coers, M., & Wtenweerde, N. (2018). Managing flexible work arrangements: Teleworking and output controls. European Management Journal, 36(6), 727–735. 10.1016/j.emj.2018.01.007

[ref18] Harrison, D.A., & Klein, K.J. (2007). What’s the difference? Diversity constructs as separation, variety, or disparity in organizations. Academy of Management Review, 32(4), 1199–1228. 10.5465/amr.2007.26586096

[ref19] Hartig, T., Kylin, C., & Johansson, G. (2007). The telework tradeoff: Stress mitigation vs. constrained restoration. Applied Psychology, 56(2), 231–253. 10.1111/j.1464-0597.2006.00252.x

[ref20] Houghton, J.D., & Neck, C.P. (2002). The revised self-leadership questionnaire: Testing a hierarchical factor structure for self-leadership. Journal of Managerial Psychology, 17(7/8), 672–691. 10.1108/02683940210450484

[ref21] Huws, U. (1996). Teleworking and Gender. Brighton, UK: The Institute for Employment Studies.

[ref22] Karia, N., & Asaari, M.H.A.H. (2016). Innovation capability: the impact of teleworking on sustainable competitive advantage. International Journal of Technology, Policy and Management, 16(2), 181–194. 10.1504/IJTPM.2016.076318

[ref23] Kato, T.A., Shinfuku, N., & Tateno, M. (2020). Internet society, internet addiction, and pathological social withdrawal: the chicken and egg dilemma for internet addiction and hikikomori. Current Opinion in Psychiatry, 33(3), 264-270. 10.1097/YCO.000000000000060 32101902

[ref24] Kerrin, M., & Hone, K. (2001). Job seekers’ perceptions of teleworking: A cognitive mapping approach. New Technology, Work and Employment, 16(2), 130–143. 10.1111/1468-005X.00082

[ref25] Kwon, M., & Jeon, S.H. (2017). Why permit telework? Exploring the determinants of California city governments’ decisions to permit telework. Public Personnel Management, 46(3), 239–262. 10.1177/0091026017717240

[ref26] Lee, S.Y., & Brand, J.L. (2005). Effects of control over office workspace on perceptions of the work environment and work outcomes. Journal of Environmental Psychology, 25(3), 323–333. 10.1016/j.jenvp.2005.08.001

[ref27] Locke, E.A., Frederick, E., Lee, C., & Bobko, P. (1984). Effect of self-efficacy, goals, and task strategies on task performance. Journal of Applied Psychology, 69(2), 241–251. 10.1037/0021-9010.69.2.241

[ref28] Locke, E.A., & Latham, G.P. (2006). New directions in goal-setting theory. Current Directions in Psychological Science, 15(5), 265–268. 10.1111/j.1467-8721.2006.00449.x

[ref29] Nakrošiene, A., Buciuniene, I., & Goštautaite, B. (2019). Working from home: Characteristics and outcomes of telework. International Journal of Manpower, 40(1), 87–101. 10.1108/IJM-07-2017-0172

[ref30] Netemeyer, R.G., Boles, J.S., & McMurrian, R. (1996). Development and validation of work–family conflict and family–work conflict scales. Journal of Applied Psychology, 81(4), 400–410. 10.1037/0021-9010.81.4.400

[ref31] Netemeyer, R.G., Maxham I, J.G., & Pullig, C. (2005). Conflicts in the work–family interface: Links to job stress, customer service employee performance, and customer purchase intent. Journal of Marketing, 69(2), 130–143. 10.1509/jmkg.69.2.130.60758

[ref32] Nohe, C., Michel, A., & Sonntag, K. (2014). Family–work conflict and job performance: A diary study of boundary conditions and mechanisms. Journal of Organizational Behavior, 35(3), 339–357. 10.1002/job.1878

[ref33] Parasuraman, S., & Simmers, C.A. (2001). Type of employment, work–family conflict and well-being: A comparative study. Journal of Organizational Behavior, 22, 551–568.

[ref34] Raišienė, A.G., Rapuano, V., Varkulevičiūtė, K., & Stachová, K. (2020). Working from home—Who is happy? A survey of Lithuania’s employees during the Covid-19 quarantine period. Sustainability, 12(13), 5332–5353. 10.3390/su12135332

[ref35] Schaufeli, W.B., Shimazu, A., Hakanen, J., Salanova, M., & De Witte, H. (2019). An ultra-short measure for work engagement. European Journal of Psychological Assessment, 35(4), 577–591. 10.1027/1015-5759/a000430

[ref36] Sener, I.N., & Bhat, C R. (2011). A copula-based sample selection model of telecommuting choice and frequency. Environment and Planning A, 43(1), 126–145. 10.1068/a43133

[ref37] Sharit, J., Czaja, S.J., Hernandez, M.A., & Nair, S.N. (2009). The employability of older workers as teleworkers: An appraisal of issues and an empirical study. Human Factors and Ergonomics in Manufacturing & Service Industries, 19(5), 457–477. 10.1002/hfm.20138 PMC2808041 20090856

[ref38] Staples, D.S., Hulland, J.S., & Higgins, C.A. (1999). A self-efficacy theory explanation for the management of remote workers in virtual organizations. Organization Science, 10(6), 758–776. 10.1287/orsc.10.6.758

[ref39] Sullivan, C. (2003). What’s in a name? Definitions and conceptualisations of teleworking and homeworking. New Technology, Work and Employment, 18(3), 158–165. 10.1111/1468-005X.00118

[ref40] Sullivan, C., & Lewis, S. (2001). Home-based telework, gender, and the synchronization of work and family: Perspectives of teleworkers and their co-residents. Gender, Work, and Organization, 8, 123–145. 10.1111/1468-0432.00125

[ref41] Toscano, F., & Zappalà, S. (2020a). Smart working in Italia: origine, diffusione e possibili esiti. Psicologia Sociale, 15(2), 203–223. 10.1482/96843

[ref42] Toscano, F., & Zappalà, S. (2020b). Social isolation and stress as predictors of productivity perception and remote work satisfaction during the COVID-19 pandemic: The role of concern about the virus in a moderated double mediation. Sustainability, 12(23), 9804. 10.3390/su12239804

[ref43] Vander Elst, T., Verhoogen, R., Sercu, M., Van den Broeck, A., Baillien, E., & Godderis, L. (2017). Not extent of telecommuting, but job characteristics as proximal predictors of work-related well-being. Journal of Occupational and Environmental Medicine, 59(10), e180-e186. 10.1097/JOM.0000000000001132 28820860

[ref44] Virick, M., DaSilva, N., & Arrington, K. (2010). Moderators of the curvilinear relation between extent of telecommuting and job and life satisfaction: The role of performance outcome orientation and worker type. Human Relations, 63(1), 137–154. 10.1177/0018726709349198

[ref45] Wang, C., Pan, R., Wan, X., Tan, Y., Xu, L., McIntyre, R. S., ... & Ho, C. (2020). A longitudinal study on the mental health of general population during the COVID-19 epidemic in China. Brain, Behavior, and Immunity, 87, 40–48. 10.1016/j.bbi.2020.04.028 32298802 PMC7153528

[ref46] Weinert, C., Maier, C., & Laumer, S. (2015). Why are teleworkers stressed? An empirical analysis of the causes of telework-enabled stress. Wirtschaftsinformatik Proceedings 2015, 1407–1421.

[ref47] Zappalà, S., Toscano, F., & Topa, G. (2021). The implementation of a remote work program in an Italian municipality before COVID-19: Suggestions to HR officers for the post-COVID-19 era. European Journal of Investigation in Health, Psychology and Education, 11(3), 866–877. 10.3390/ejihpe11030064 34563077 PMC8544214

[ref48] Zhang, S., Moeckel, R., Moreno, A. T., Shuai, B., & Gao, J. (2020). A work-life conflict perspective on telework. Transportation Research Part A: Policy and Practice, 141, 51–68. 10.1016/j.tra.2020.09.007 32982087 PMC7509537

